# Immune Mechanisms Underlying Neonatal Protection Following Maternal RSV Vaccination

**DOI:** 10.3390/ijms27146363

**Published:** 2026-07-17

**Authors:** Aikaterini I. Nikolaou, Vasileios Giapros, Maria Alexandra Kefala, Nikolaos G. Papanikolaou, Maria Baltogianni, Fani Ladomenou

**Affiliations:** 1 Department of Pediatrics, School of Medicine, University of Ioannina, 45500 Ioannina, Greece; nikaikaterini@gmail.com (A.I.N.); fladomenou@gmail.com (F.L.); 2 Neonatal Intensive Care Unit, School of Medicine, University of Ioannina, 45500 Ioannina, Greece; mariakefala2004@gmail.com (M.A.K.); mbalt@doctors.org.uk (M.B.); 3 School of Medicine, Faculty of Health Sciences, National and Kapodistrian University of Athens, 11527 Athens, Greece; smd2300123@uoa.gr

**Keywords:** respiratory syncytial virus, maternal immunization, transplacental antibody transfer, neonatal immunity, FcRn, antibody glycosylation, IgG, Fc-mediated effector functions, passive immunity, vaccination timing

## Abstract

Respiratory syncytial virus (RSV) remains a major cause of severe lower respiratory tract infection in early infancy, a period characterized by immunological immaturity and limited capacity for effective antiviral responses. Maternal RSV vaccination has emerged as a successful strategy to protect newborns by inducing high concentrations of IgG1-dominant, prefusion F-specific antibodies, which are selectively and actively transported across the placenta. This review synthesizes current mechanistic insights into how maternally derived antibodies confer neonatal protection, focusing on (i) FcRn-mediated transplacental transport, (ii) IgG subclass-specific differences in transfer and half-life, and (iii) the role of Fc glycosylation in modulating Fcγ receptor engagement and effector functions. Beyond neutralization, vaccine-induced antibodies mediate Fc-dependent mechanisms such as antibody-dependent cellular cytotoxicity and phagocytosis, which may be preferentially enriched in the neonatal circulation. Although emerging systems serology data suggest qualitative selectivity in placental transfer, evidence remains heterogeneous and highlights the need for further clarification of glycosylation-dependent and glycosylation-independent pathways. The timing of vaccination, maternal antibody characteristics, and placental integrity critically influence neonatal antibody levels and the duration of passive immunity. Understanding these molecular determinants is essential for optimizing maternal RSV immunization strategies and improving early-life protection.

## 1. Introduction

Respiratory syncytial virus (RSV) is the leading cause of acute lower respiratory tract infection (LRTI) in infants worldwide and continues to account for a substantial proportion of pediatric hospitalizations and healthcare utilization [1,2,3]. Disease burden is greatest during the first months of life, when the functional immaturity of both innate and adaptive immunity limits the infant’s ability to mount effective immune responses [4,5]. As a result, preventing severe RSV disease during this vulnerable period has become a major priority for pediatric healthcare systems worldwide.

The development of RSV vaccines has long been hindered by safety concerns following the formalin-inactivated RSV vaccine trials of the 1960s, which resulted in vaccine-enhanced respiratory disease [6]. The introduction of structure-based vaccine design, particularly stabilization of the RSV fusion (F) glycoprotein in its prefusion conformation, marked a major breakthrough by preserving highly neutralization-sensitive epitopes and substantially improving vaccine immunogenicity [2,6,7,8,9]. These advances ultimately enabled the development of maternal RSV vaccines designed to protect infants through placental transfer of vaccine-induced antibodies.

Maternal immunization provides an opportunity to protect infants during the period of highest susceptibility to severe RSV disease. Vaccination during pregnancy induces pathogen-specific maternal IgG antibodies that are actively transferred across the placenta, providing passive immunity before the infant develops mature adaptive immune responses [10,11,12,13,14,15,16]. Long-acting monoclonal antibodies, particularly nirsevimab, represent an alternative preventive approach by providing immediate passive protection directly to the infant after birth [17]. Clinical trials and real-world studies have demonstrated high effectiveness for both interventions in reducing RSV-associated hospitalization and severe lower respiratory tract disease, although comparative studies have suggested greater effectiveness of monoclonal antibodies in selected clinical settings [17,18,19,20].

Although both preventive strategies effectively reduce the burden of severe RSV disease by providing passive antibody-mediated protection to the infant, they differ in the origin, generation, and delivery of the protective antibodies. Maternal vaccination induces a polyclonal antibody response in the mother, followed by transplacental transfer of vaccine-induced antibodies to the fetus, whereas monoclonal antibody administration provides a predefined antibody directly to the infant, independently of maternal immune activation and placental transfer. Understanding the mechanisms that govern maternal antibody generation, placental transfer, and neonatal antibody function is therefore essential for optimizing maternal immunization strategies and identifying the determinants of effective neonatal protection.

While neutralizing antibodies remain the best-established correlate of protection following maternal RSV vaccination, accumulating evidence suggests that qualitative antibody characteristics—including IgG subclass distribution, Fc receptor interactions, and Fc glycosylation patterns—may also influence antibody transfer, persistence, and functional activity [21,22,23,24,25,26]. These features have been associated with differences in placental transfer efficiency and Fc-mediated effector functions, including antibody-dependent cellular cytotoxicity (ADCC), antibody-dependent cellular phagocytosis (ADCP), and complement activation. However, current evidence remains heterogeneous, and the relative contribution of these qualitative antibody characteristics to the clinical protection observed following maternal RSV vaccination has not yet been fully established. Further mechanistic and clinical studies are needed to determine how these qualitative antibody characteristics contribute to neonatal protection and whether they represent meaningful correlates of vaccine-induced immunity.

Against this evolving preventive landscape, this narrative review examines the immune mechanisms underlying neonatal protection following maternal RSV vaccination. Particular attention is given to FcRn-mediated antibody transfer, IgG subclass distribution, Fc glycosylation, and Fc-dependent antibody functions, while critically discussing the current evidence supporting these mechanisms. The review also examines the complementary roles of maternal vaccination and long-acting monoclonal antibodies and highlights key areas where further mechanistic and clinical research is needed.

## 2. Literature Search Methodology

A structured literature search was conducted between March and June 2026 to identify studies investigating maternal immunization against RSV, transplacental antibody transfer, and neonatal immune protection. This review was designed as a structured narrative review rather than a formal systematic review; therefore, no predefined protocol or comprehensive systematic screening strategy was applied.

Two independent reviewers searched the PubMed and Google Scholar databases using combinations of the following keywords: (“respiratory syncytial virus” OR RSV) AND (“maternal vaccination” OR “maternal immunization”) AND (“placental transfer” OR “transplacental antibody transfer” OR FcRn) AND (“neonate” OR “newborn” OR “infant”). Eligible studies included human clinical trials, observational studies, cohort studies, and selected experimental studies, providing data on maternal antibody responses, placental transfer mechanisms, antibody functionality, or neonatal clinical outcomes, as well as studies addressing relevant molecular and immunological mechanisms such as Fc receptor biology and antibody glycosylation. Only articles published in English were considered. Studies focusing exclusively on adult populations without relevance to maternal–fetal immunity, as well as non-translational animal studies, were excluded. Studies published from database inception until June 2026 were considered.

**Figure 1:**
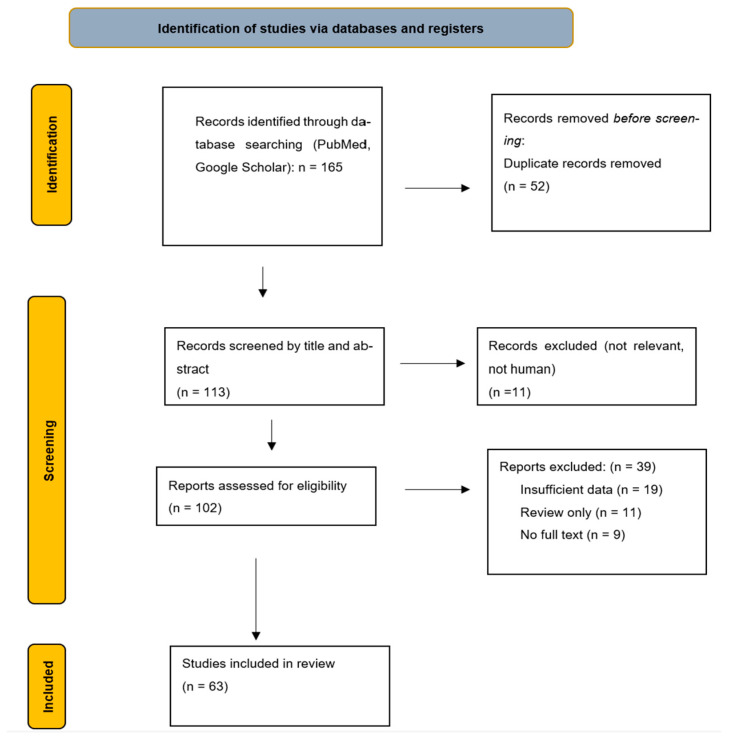
PRISMA flow diagram of search procedure.

Titles and abstracts were screened to identify relevant publications, followed by full-text evaluation of selected articles. Study inclusion was based on relevance to the scope of the review rather than strict systematic inclusion criteria, and any disagreements between reviewers were resolved through discussion and consensus. A total of 165 records were initially identified, of which 63 studies were ultimately included. Details of the literature search and study selection process are presented in the PRISMA flow diagram (Figure 1). As this is a structured narrative review, no formal assessment of study quality or risk of bias was performed, which represents a limitation of the present work. The search strategy was designed to ensure broad coverage of the available literature rather than exhaustive systematic inclusion.

## 3. RSV Structure and Vaccine Targets

RSV is an enveloped, negative-sense, single-stranded RNA virus belonging to the family *Pneumoviridae* and the genus *Orthopneumovirus*. The viral genome encodes multiple structural and nonstructural proteins that contribute to viral replication, host cell entry, and immune evasion. Among these, the surface glycoproteins—the fusion (F) protein and the attachment (G) protein—play central roles in viral infectivity and constitute the primary targets of the host humoral immune response [6]. The Fc glycosylation profile of maternal IgG influences antibody effector functions and is associated with selective placental transfer to the fetus [27]. Serological assays targeting antibodies against RSV surface glycoproteins are widely used to evaluate immune responses following natural infection and vaccination [28].

The RSV G glycoprotein mediates viral attachment to host epithelial cells by facilitating interactions with cellular receptors on the respiratory epithelium. However, the G protein exhibits substantial antigenic variability across viral strains, limiting its utility as a stable vaccine antigen [6]. In contrast, the RSV F protein is highly conserved and is essential for mediating the fusion of the viral envelope with the host cell membrane, a critical step for viral entry. This process involves conformational rearrangements that enable insertion of the fusion peptide into the host membrane, ultimately driving membrane merger and viral entry into the cytoplasm [2,8].

The F glycoprotein exists in two distinct conformational states during the viral lifecycle: a metastable prefusion conformation (preF) and a highly stable postfusion conformation (postF). Upon triggering, the prefusion F undergoes an irreversible structural transition to the postfusion state, accompanied by extensive refolding of heptad repeat regions that drive membrane fusion [8]. Structural studies have demonstrated that the prefusion conformation exposes multiple highly neutralization-sensitive antigenic epitopes that are either absent or conformationally altered in the postfusion structure. Among these, antigenic site Ø and antigenic site V are key targets of the most potent neutralizing antibodies generated following natural infection [2,6,8].

Historically, early RSV vaccine candidates based on postfusion F antigens were capable of inducing binding antibody responses but elicited relatively low levels of neutralizing activity, highlighting the importance of antigen conformation in shaping protective immunity [6]. Advances in structural vaccinology enabled the rational stabilization of the prefusion F conformation through engineered mutations that prevent spontaneous refolding into the postfusion state. These stabilized preF immunogens preserve conformational epitopes associated with high neutralizing potency and have been shown to induce substantially higher neutralizing antibody titers compared with postfusion-based vaccines [6,8].

Recent experimental studies indicate that antibodies targeting the prefusion F protein may exhibit Fc-dependent functional properties in addition to neutralizing activity, including enhanced engagement of Fcγ receptors and induction of effector mechanisms such as ADCC and antibody-dependent cellular phagocytosis [21,23,29]. Although these observations are biologically plausible, their contribution to the clinical protection achieved by maternal RSV vaccination has not yet been fully established.

These structural and immunological insights have directly informed the design of current RSV vaccine strategies, including vaccines developed for maternal immunization. The bivalent prefusion F protein vaccine administered during pregnancy induces robust maternal antibody responses targeting conserved neutralizing epitopes of the RSV F protein. These antibodies are subsequently transferred across the placenta via FcRn-mediated transport, providing passive protection to the fetus during a critical window of vulnerability in early life [13,18].

The structural characterization of the RSV F glycoprotein has fundamentally changed vaccine development by enabling the design of prefusion F-based immunogens capable of eliciting potent neutralizing antibody responses. These advances provide the biological basis for current maternal RSV vaccination strategies and underpin efforts to improve protection against severe RSV disease during early infancy.

## 4. Immunological Basis of Maternal Vaccination and Passive Neonatal Immunity

The neonatal immune system is functionally immature and undergoes rapid development during the first months of life. Several characteristics of neonatal immunity contribute to increased susceptibility to respiratory viral infections, including reduced antigen-presenting cell function, limited germinal center formation, and a bias toward T helper 2 (Th2)-skewed immune responses. In addition, the capacity to generate high-affinity antibody responses and durable immunological memory is not fully established in early infancy, thereby limiting the effectiveness of active immunization during this period [4,5,30]. For this reason, passive immunity mediated by maternally derived antibodies represents a key protective mechanism during early life [10,16,31].

Beyond direct pathogen neutralization, maternally transferred antibodies also contribute to the functional shaping of the neonatal immune system. There is evidence that maternal antibodies can modulate antigen presentation, influence B-cell maturation, and regulate immune activation thresholds in the neonate [11,16]. Systems-level analyses have demonstrated that newborns acquire a diverse repertoire of maternally derived antiviral IgG antibodies through transplacental transfer, reflecting maternal immune history and contributing to early-life protection against multiple viral pathogens [32].

Maternal immunization represents an effective strategy to enhance passive neonatal protection by increasing antigen-specific antibody concentrations in maternal circulation during pregnancy [10,11,12,28]. Vaccination induces antigen-specific B-cell activation, affinity maturation, and class switching, resulting in the production of high-affinity IgG antibodies directed against pathogen-specific antigens. These antibodies are actively transported to the fetus via receptor-mediated mechanisms primarily involving the FcRn, thereby conferring passive immunity during a period when endogenous immune responses remain immature [13,14,24,33]. Experimental studies further support this mechanism, demonstrating that vaccine-induced RSV-specific antibodies generated during pregnancy are efficiently transferred to offspring, with neonatal antibody levels closely correlating with maternal titers [34].

Among human immunoglobulin isotypes, IgG is uniquely capable of efficient transplacental transfer. IgG comprises four subclasses—IgG1, IgG2, IgG3, and IgG4—which differ in their transfer efficiency. IgG1 is transferred most efficiently, followed by IgG3 and IgG4, whereas IgG2 demonstrates relatively lower transfer efficiency [3,4,14]. These differences are associated with subclass-specific variations in FcRn binding affinity and structural properties of the Fc domain, which influence receptor engagement and intracellular trafficking during transcytosis [13,24,33]. Consequently, immune responses directed against protein antigens, such as viral glycoproteins, typically induce IgG1-dominated responses that are optimally suited for placental transfer and neonatal protection.

The efficiency of maternal antibody transfer increases progressively throughout gestation, with the highest transfer occurring during the third trimester. This pattern reflects both increased FcRn expression and the maturation of placental transport pathways within syncytiotrophoblast cells [3,14,24]. Evidence from non-human primate models demonstrates efficient transplacental transfer of RSV-specific IgG, with neonatal antibody levels at birth closely mirroring maternal concentrations [35]. Conversely, preterm infants receive significantly lower levels of maternally derived antibodies due to reduced duration of exposure to placental transfer mechanisms, contributing to increased susceptibility to severe infections [36,37,38,39]. Additional factors, including maternal hypergammaglobulinemia and placental pathology, have also been shown to impair antibody transfer efficiency [37,40,41].

Maternally derived antibodies mediate protection against viral infections through both neutralizing and Fc-dependent mechanisms. Neutralizing antibodies targeting the RSV fusion (F) glycoprotein are a key correlate of protection and function by preventing viral entry into host cells [6]. In parallel, Fc-dependent effector functions—including ADCC, ADCP, and complement activation—are mediated through interactions between the Fc region of IgG and FcγRs expressed on immune effector cells such as natural killer cells, macrophages, and neutrophils [5,22,42]. Experimental evidence suggests that these mechanisms may contribute to the clearance of virus-infected cells and the amplification of antiviral immune responses, although their specific contribution to the clinical protection achieved following maternal RSV vaccination remains incompletely defined.

In addition to transplacental transfer, maternal immune protection may continue after birth through breastfeeding. Breast milk contains RSV-specific antibodies together with cytokines, immune cells, human milk oligosaccharides, and other bioactive components that support mucosal immunity and early immune development [43]. However, the extent to which breastfeeding-derived antibodies contribute to protection against RSV disease remains to be fully established. The effectiveness of maternally derived IgG is also influenced by its structural characteristics, including subclass distribution and Fc glycosylation patterns. These properties affect Fc receptor binding, antibody stability, and downstream immune activation, thereby potentially influencing antibody function after transfer to the infant [15,19,24,38].

The duration of passive immunity in early life is largely determined by the half-life of maternally derived IgG antibodies in the infant circulation. Following birth, antibody levels decline progressively due to physiological catabolism, with an estimated half-life of approximately three to four weeks [10,31]. Cohort studies have demonstrated that RSV-specific IgG levels decrease substantially within the first months of life, with markedly reduced concentrations observed by approximately six months of age [30,44].

Despite this progressive decline, maternally derived antibodies provide protection during the first months of life, corresponding to the period of highest susceptibility to severe RSV disease. Maternal immunization therefore aims to maximize antibody concentrations at birth and extend passive protection throughout early infancy [18,30,45].

A detailed understanding of maternal antibody responses and passive neonatal immunity is essential for further optimizing RSV prevention strategies. Current maternal RSV vaccines are designed to induce high levels of neutralizing antibodies against the prefusion F glycoprotein, which are subsequently transferred to the fetus through the placenta. Clinical studies have demonstrated robust IgG and neutralizing antibody responses following vaccination during pregnancy, supporting the effectiveness of this approach [18,46,47]. The molecular mechanisms underlying these processes are discussed in the following sections.

## 5. Mechanisms of Transplacental Antibody Transfer

Maternal antibodies transferred across the placenta constitute a critical mechanism of protection against RSV infection during early infancy, a period during which the neonatal immune system is not fully capable of mounting effective antiviral responses [5,31]. This process enables the fetus to acquire antigen-specific humoral immunity prior to birth and provides passive immune protection during the early postnatal period. Transplacental antibody transfer is an active, highly regulated, receptor-mediated process that occurs predominantly during the second and third trimesters of pregnancy and is primarily mediated by the FcRn, which is expressed on placental syncytiotrophoblast cells [13,14,24,33].

### 5.1. FcRn-Mediated IgG Transport

FcRn is a major histocompatibility complex (MHC) class I-related molecule that plays a central role in IgG homeostasis and transplacental transport [13,48]. In the placenta, FcRn is highly expressed in syncytiotrophoblast cells that form the primary interface between maternal blood in the intervillous space and the fetal circulation. IgG transfer across this barrier occurs via receptor-mediated endocytosis followed by intracellular trafficking and transcytosis.

Maternal IgG is internalized into syncytiotrophoblast cells through clathrin-mediated endocytosis, a process that is largely non-specific at the level of uptake [14,33]. Following internalization, IgG is trafficked into early endosomal compartments, where the acidic pH (~6.0–6.5) promotes high-affinity binding between the Fc region of IgG and FcRn [13,15,48]. This pH-dependent interaction is critical for protecting IgG from lysosomal degradation and directing it toward recycling or transcytotic pathways [13,48,49].

Intracellular trafficking of FcRn–IgG complexes is tightly regulated by endosomal sorting mechanisms. Additional proteins, including the multiligand receptor megalin, have been proposed to facilitate vesicular transport and receptor recycling during transcytosis, although their precise contribution to placental IgG transfer remains incompletely defined [50]. Together, these observations suggest that FcRn-mediated IgG transport depends on coordinated intracellular trafficking mechanisms rather than receptor binding alone.

Following endosomal sorting, FcRn–IgG complexes are transported toward the basolateral (fetal-facing) membrane. Upon exposure to physiological pH (~7.4), the affinity of FcRn for IgG decreases, resulting in dissociation of the complex and release of IgG into the fetal circulation [13,14]. This pH-dependent binding and release mechanism ensures directional transport of IgG across the placental barrier while preserving antibody integrity.

**Figure 2:**
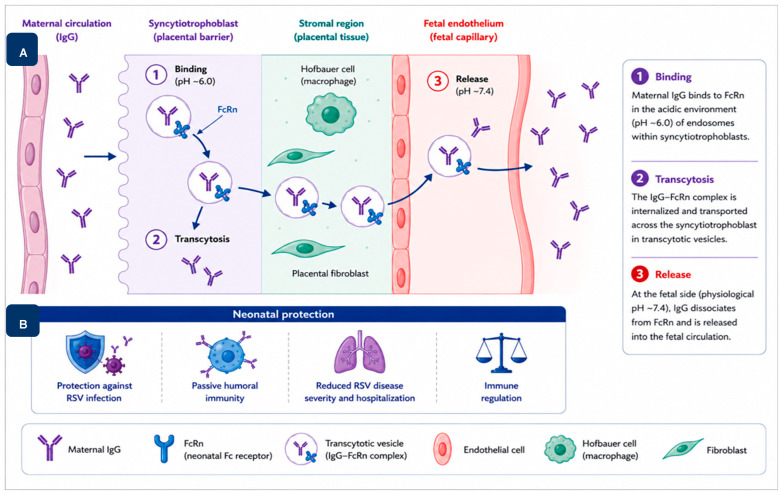
Mechanisms of maternal antibody transfer across the placenta and neonatal protection. (**A**) FcRn-mediated transplacental transfer of maternal IgG. Maternal IgG binds to the neonatal Fc receptor (FcRn) within acidic endosomal compartments of syncytiotrophoblasts, undergoes transcytosis across the placental barrier, and is released into the fetal circulation at physiological pH. (**B**) Potential contributions of maternally transferred IgG to neonatal protection, including passive humoral immunity, protection against RSV infection, reduced RSV disease severity and hospitalization, and immune regulation [13,14,33,51]. Original schematic illustration prepared specifically for this manuscript by the authors and not adapted or reproduced from any previously published source.

Notably, FcRn-mediated transport is highly selective at the level of immunoglobulin class. IgG is the only antibody isotype efficiently transported across the placenta, whereas IgM and IgA are largely excluded due to their structural characteristics and lack of FcRn interaction [14,30]. This selective transport explains why maternally derived IgG represents the dominant source of humoral immunity during fetal life and early infancy. The mechanism of FcRn-mediated transplacental IgG transport is illustrated in Figure 2.

### 5.2. Determinants of Placental Transfer Efficiency

The efficiency of transplacental IgG transfer is influenced by multiple biological and physiological factors, including gestational age, maternal antibody concentration, IgG subclass distribution, placental integrity, and maternal health status [24,33,41,51].

Gestational age represents one of the most critical determinants of transfer efficiency. IgG transfer begins during the second trimester but increases substantially during the third trimester, reflecting both increased FcRn expression and expansion of placental surface area [14,24,52]. Consequently, preterm infants receive significantly lower levels of maternally derived antibodies compared with term infants, contributing to increased susceptibility to severe infections [36,37,38,39].

Maternal antibody concentration is another key determinant of neonatal antibody levels at birth. Strong correlations between maternal serum antibody titers and cord blood concentrations have been consistently observed, indicating that higher maternal IgG levels promote more efficient transplacental transfer [24,44,53]. These observations provide the rationale for maternal vaccination strategies designed to increase maternal antibody concentrations during pregnancy and, consequently, enhance neonatal antibody levels at birth [18,45].

IgG subclass distribution further modulates transfer efficiency. IgG1 is transferred most efficiently, followed by IgG3 and IgG4, whereas IgG2 exhibits reduced transfer [4,14]. These differences are associated with subclass-specific structural properties of the Fc domain and variations in FcRn binding affinity and stability within endosomal compartments [13,24,33]. Although IgG3 displays high affinity for Fcγ receptors and potent effector functions, its relatively short serum half-life compared to IgG1 may limit its contribution to sustained neonatal protection, thereby supporting IgG1 as the predominant subclass transferred following maternal immunization [13,16].

Additional factors influencing transfer include maternal infections, placental inflammation, and systemic immunological conditions. For example, maternal hypergammaglobulinemia and chronic infections have been associated with impaired transfer efficiency, potentially due to competition for FcRn binding or altered placental function [37,40,41]. Collectively, these factors illustrate that efficient antibody transfer depends on both maternal and placental characteristics rather than FcRn expression alone. The principal determinants of transplacental antibody transfer are summarized in Table 1.

### 5.3. Selective Transfer of Functional Antibodies

While early models of maternal–fetal antibody transfer emphasized FcRn-mediated transport as a largely quantitative process, accumulating evidence indicates that placental transfer may exhibit qualitative selectivity. Systems serology studies have demonstrated that antibodies transferred to the fetus differ functionally from those present in maternal circulation, suggesting preferential transfer of antibodies with specific Fc-mediated properties [26,38,54].

In particular, antibodies enriched in cord blood have been shown to display enhanced capacity to engage Fcγ receptors and activate innate immune effector cells, including natural killer cells and phagocytes [25]. These antibodies are associated with Fc glycan profiles characterized by increased galactosylation and other structural modifications that may enhance Fc receptor binding and functional activity [21,25].

However, evidence regarding glycosylation-dependent selectivity remains complex. Earlier studies have reported broadly similar Fc glycosylation profiles between maternal and fetal IgG, suggesting that FcRn binding itself is largely independent of glycosylation and that additional regulatory mechanisms may contribute to selective transfer [27]. More recent data indicate that Fc glycosylation may indirectly influence transfer by modulating interactions with Fc receptors or affecting antibody structure and stability [15,24].

Functionally, maternally derived antibodies contribute to neonatal protection through both neutralizing activity and Fc-mediated effector mechanisms. Systems serology studies suggest that preferential transfer of antibodies with enhanced Fc functionality may contribute to neonatal immune protection beyond viral neutralization. However, the clinical significance of this proposed selective transfer has not yet been fully established.

## 6. Antibody Glycosylation and Its Role in Placental Transfer and Neonatal Immunity

IgG antibodies are glycoproteins whose biological activity is strongly influenced by post-translational glycosylation of the Fc region. Each IgG molecule contains a conserved N-linked glycosylation site at asparagine 297 (Asn297) within the CH2 domain of the Fc region, where complex biantennary glycans are attached [21,24]. These glycans consist of a core structure composed of N-acetylglucosamine (GlcNAc) and mannose residues and can undergo additional modifications, including galactosylation, sialylation, fucosylation, and the incorporation of bisecting GlcNAc. Variations in Fc glycan composition can modulate the structural conformation and flexibility of the Fc domain, thereby influencing interactions with Fc receptors, complement components, and other immune mediators [15,21]. Therefore, Fc glycosylation plays a central role in regulating antibody effector function, half-life, and may indirectly influence transplacental antibody transfer [24].

Glycosylation patterns of circulating IgG antibodies are dynamic and are influenced by physiological and immunological factors, including age, sex, infection, inflammation, and vaccination [21]. Pregnancy is associated with distinct and coordinated alterations in antibody glycosylation, reflecting the immunomodulatory adaptations required to maintain maternal–fetal tolerance [15,24]. These changes may affect both maternal immune responses and the efficiency and quality of antibody transfer to the fetus.

### 6.1. Structural Diversity of IgG Fc Glycans

The Fc-associated glycan is essential for maintaining both the structural integrity and biological activity of IgG antibodies. In its canonical form, the Fc glycan consists of a complex biantennary structure that occupies a central position between the CH2 domains of the Fc region, contributing to Fc domain stability and receptor accessibility [21].

Terminal modifications of this glycan critically influence antibody function. Galactosylation refers to the addition of galactose residues to the terminal branches, whereas sialylation involves the attachment of sialic acid to galactose moieties. Core fucosylation involves the addition of fucose to the innermost GlcNAc residue of the glycan core. These modifications alter Fc conformational dynamics and modulate interactions with FcγRs expressed on innate immune cells such as natural killer (NK) cells, macrophages, and neutrophils [21,24].

Because Fcγ receptor engagement is central to antibody-mediated immune function, variation in glycosylation patterns can substantially alter the effector profile of antibodies generated during infection or vaccination [22,23].

### 6.2. Glycosylation Patterns During Pregnancy

Pregnancy induces characteristic changes in IgG glycosylation profiles that are thought to contribute to immune regulation at the maternal–fetal interface. Several studies have demonstrated increased levels of galactosylation and sialylation during gestation, which are associated with anti-inflammatory antibody profiles and reduced pro-inflammatory Fc-mediated activity [15,21,24].

These glycosylation changes are thought to contribute to immune tolerance while preserving protective immunity. They may also influence the functional properties of antibodies transferred to the fetus, although the extent of their contribution remains under investigation. However, their impact extends beyond maternal immune modulation, as they may also influence the functional properties of antibodies transferred to the fetus. Antibodies enriched in galactosylated and sialylated glycans have been associated with altered Fc receptor binding profiles and may exhibit enhanced or modulated effector functions depending on the immunological context [21,24].

### 6.3. Glycosylation and Selective Placental Antibody Transfer

Recent studies indicate that antibody glycosylation may contribute to the qualitative features of placental antibody transfer. Importantly, FcRn-mediated IgG binding and transcytosis are largely independent of Fc glycosylation. Instead, glycosylation is thought to influence antibody function indirectly by modulating Fcγ receptor binding, molecular stability, and intracellular trafficking pathways, which may ultimately affect the quality of antibodies transferred to the fetus [13,15].

Systems serology studies have demonstrated that antibodies transferred to the fetus can exhibit distinct Fc profiles compared with those in maternal circulation, indicating a degree of functional selection during transplacental transport [25,26]. In particular, antibodies enriched in digalactosylated Fc glycans have been associated with enhanced binding to FcγRIIIa and improved capacity to activate natural killer cells, suggesting that functionally potent antibodies may be preferentially transferred [25].

However, the extent and consistency of this selective transfer remain a subject of ongoing investigation. Earlier studies have reported largely comparable Fc glycosylation profiles between maternal and cord blood IgG, suggesting limited or no strong glycan-dependent selection during FcRn-mediated transport [27]. Moreover, findings across studies are not fully consistent, likely reflecting differences in study design, population characteristics, and analytical approaches. These discrepancies indicate that selective transfer of functional antibodies is context-dependent and not yet fully defined.

Taken together, current evidence suggests that placental antibody transfer may be influenced by both quantitative and qualitative factors. However, the relative contribution of these mechanisms to neonatal protection following maternal RSV vaccination remains uncertain and requires further mechanistic investigation.

### 6.4. Glycosylation and Fc-Dependent Effector Functions

Fc glycosylation plays a critical role in regulating antibody effector functions through modulation of Fc receptor interactions. It is important to note that Fc glycosylation does not directly determine FcRn binding affinity. Instead, glycosylation primarily affects antibody function by influencing Fcγ receptor binding, molecular stability, and the inflammatory profile of the antibody response. One of the most well-characterized examples is the effect of core fucosylation on FcγRIIIa binding. Afucosylated IgG exhibits increased affinity for FcγRIIIa on NK cells, resulting in enhanced ADCC [21,23].

**Figure 3:**
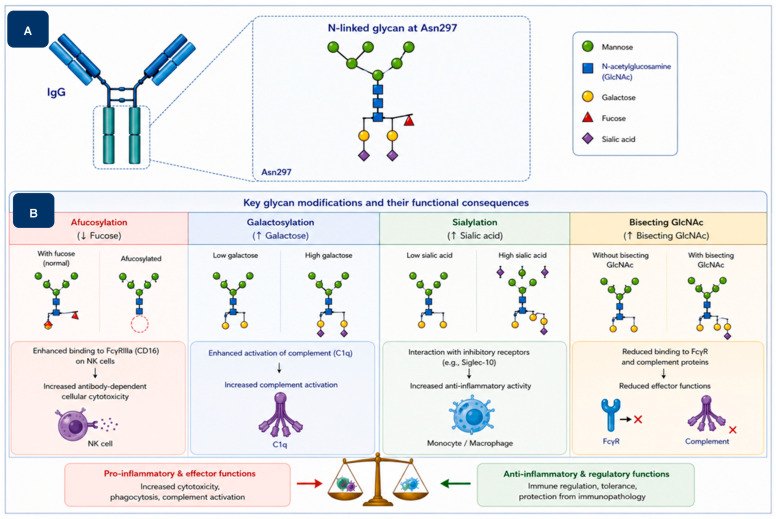
Influence of IgG Fc glycosylation on antibody effector functions. (**A**) Schematic representation of the conserved N-linked glycan at Asn297 of the IgG Fc domain. (**B**) Key Fc glycan modifications, including changes in fucosylation, galactosylation, sialylation, and bisecting N-acetylglucosamine (GlcNAc), and their potential effects on Fc receptor engagement, complement activation, and downstream inflammatory, effector, or regulatory antibody functions [15,21,24,25,27]. Original schematic illustration prepared specifically for this manuscript by the authors and not adapted or reproduced from any previously published source.

Similarly, galactosylation has been associated with increased complement activation through enhanced C1q binding, whereas sialylation has been linked to immunomodulatory and anti-inflammatory effects, potentially through altered Fc receptor engagement [21,22]. These glycan-dependent modifications collectively influence the balance between pro-inflammatory and regulatory immune responses. The major Fc glycosylation modifications and their functional immunological consequences are summarized in Table 2. The impact of Fc glycosylation on antibody effector functions is summarized in Figure 3.

### 6.5. Implications for Maternal RSV Vaccination

Emerging evidence suggests that Fc glycosylation influences antibody function and may contribute to the qualitative characteristics of placental antibody transfer, although the clinical relevance of these observations remains to be established [15,21,24,25,26,27]. Vaccine-induced antibodies are defined not only by their antigen specificity and concentration but also by their structural and functional properties, including Fc glycan composition [15,21,24].

Maternal RSV vaccination is designed to induce high titers of neutralizing antibodies directed against the prefusion F glycoprotein. In addition to neutralizing activity, experimental evidence suggests that the biological properties of these antibodies, including their capacity to engage Fc receptors, may also influence neonatal immune protection following placental transfer. However, the clinical relevance of these qualitative antibody characteristics has not yet been fully established [21,54].

Future research integrating structural immunology, systems serology, and maternal–fetal immunology will be essential to elucidate how vaccine-induced antibody features influence placental transfer and early-life immunity. A deeper understanding of these mechanisms may guide the development of next-generation maternal vaccines capable of eliciting antibody responses that are not only quantitatively robust but also qualitatively optimized for neonatal protection.

## 7. Functional Mechanisms of Neonatal Protection Mediated by Maternal Antibodies

Maternally derived antibodies transferred across the placenta play a central role in protecting infants against RSV during early life, primarily through viral neutralization, while Fc-dependent effector functions may provide additional protection [4,5,13,24,31,55]. These complementary mechanisms are discussed in the following sections.

### 7.1. Neutralizing Antibody Responses

Neutralizing antibodies represent a primary correlate of protection against RSV infection and function by blocking viral entry into host cells. These antibodies target viral surface glycoproteins, particularly the fusion (F) protein, and inhibit the conformational changes required for membrane fusion and viral entry into respiratory epithelial cells [6,8].

The prefusion conformation of the RSV F protein exposes highly neutralization-sensitive epitopes, including antigenic sites Ø and V, which are the targets of the most potent neutralizing antibodies generated following infection or vaccination [6,8,56]. Maternal immunization with prefusion-stabilized RSV F protein vaccines induces high titers of such antibodies, which are efficiently transferred to the fetus and provide protection during early infancy. Clinical evidence demonstrates that these maternally derived antibodies significantly reduce the incidence of RSV-associated lower respiratory tract disease and hospitalization in infants during the first months of life [18].

Although neutralizing antibodies represent the principal correlate of protection, experimental and systems serology studies suggest that Fc-mediated antibody functions may also contribute to antiviral immunity [5,54].

### 7.2. Fc-Dependent Effector Functions

In addition to neutralizing viral particles, IgG antibodies mediate protection through Fc-dependent effector mechanisms involving interactions with FcγRs expressed on innate immune cells [22,42]. These interactions require Fc receptor crosslinking and initiate intracellular signaling cascades that lead to immune cell activation and effector function [22,57].

ADCC is a key mechanism in which IgG antibodies bind to viral antigens expressed on the surface of infected cells. The Fc region of these antibodies engages FcγRIIIa receptors on natural killer (NK) cells, triggering degranulation and release of cytotoxic mediators such as perforin and granzymes, leading to targeted elimination of infected cells [22,57].

ADCP represents another critical pathway, whereby antibody-opsonized viral particles or infected cells are recognized by Fcγ receptors on phagocytic cells, including macrophages and neutrophils, resulting in internalization and degradation of the target [5,23]. In parallel, antibodies can activate the classical complement pathway through C1q binding, promoting opsonization and further enhancing phagocytic clearance [22].

These Fc-mediated mechanisms extend antibody function beyond viral neutralization and may contribute to coordinated activation of innate immune responses.

### 7.3. Functional Antibody Profiles in Early Life

Advances in systems serology have revealed that maternally transferred antibodies exhibit diverse functional properties that influence neonatal immunity. Comparative analyses of maternal and cord blood antibody repertoires have demonstrated that antibodies present in neonatal circulation can differ functionally from those in maternal serum, suggesting selective enrichment of specific antibody features during placental transfer [25,26,38].

In particular, antibodies with enhanced capacity to engage Fcγ receptors associated with natural killer cell activation and phagocytosis have been observed in cord blood [25]. These findings support the concept that the placenta may preferentially transfer antibodies with increased functional potential, including the ability to mediate Fc-dependent effector responses. These observations support the hypothesis that placental transfer may favor antibodies with distinct functional characteristics.

### 7.4. Integration of Neutralizing and Fc-Mediated Protection

Protection against RSV during early infancy is likely to result from the combined activity of neutralizing antibodies and additional Fc-mediated immune mechanisms. Evidence from human studies further supports the role of antibody-mediated protection, as higher levels of RSV-specific antibodies—including those present in breast milk—have been associated with reduced risk of RSV-associated illness in infants [43]. Neutralizing antibodies prevent viral entry and limit initial infection, whereas Fc-mediated pathways promote clearance of infected cells and amplify antiviral immune responses.

IgG1 antibodies, which are preferentially transferred across the placenta, exhibit strong capacity to engage Fcγ receptors and mediate effector activity [14,24]. In addition, specific glycosylation profiles can modulate Fc receptor binding affinity and downstream immune activation, thereby influencing the overall functional potency of transferred antibodies [21,25].

Overall, current evidence indicates that maternally derived antibodies protect newborns primarily through viral neutralization, whereas Fc-mediated immune mechanisms may provide additional protection. However, the relative contribution of these mechanisms to the clinical efficacy of maternal RSV vaccination remains to be established.

## 8. Timing of Maternal Vaccination and Determinants of Antibody Transfer

The effectiveness of maternal RSV vaccination depends not only on the magnitude and functional characteristics of vaccine-induced antibodies but also on the timing of immunization during pregnancy. The interval between vaccination and delivery influences maternal antibody levels, placental antibody transfer, and ultimately the degree of passive immunity acquired by the newborn at birth [18,54]. Appropriate vaccination timing is therefore essential to maximize neonatal protection during the period of greatest susceptibility to severe RSV disease.

### 8.1. Gestational Timing of Antibody Transfer

The transfer of maternal IgG across the placenta increases progressively throughout gestation and becomes most efficient during the third trimester [14,16,31,57]. This pattern reflects both structural and functional maturation of the placenta, including increased surface area and enhanced expression of FcRn on syncytiotrophoblast cells, which mediate receptor-dependent transcytosis of IgG [13,14,24,33].

Although IgG transfer begins during the second trimester, the majority of maternal antibodies are transferred during late gestation, when FcRn-mediated transport reaches maximal efficiency [30,33]. As a result, infants born prematurely receive substantially lower levels of maternally derived antibodies compared with term infants [24,41]. Observational studies have consistently demonstrated that prematurity and low birthweight are associated with reduced cord blood antibody concentrations across multiple pathogens, including RSV and influenza [36,37,53].

In the context of RSV, studies evaluating maternal–infant pairs have shown that preterm birth is associated with lower cord blood RSV-specific IgG levels and reduced cord-to-maternal transfer ratios compared with full-term delivery [39,53]. These findings underscore the critical role of gestational age in determining the efficiency of passive antibody transfer and neonatal immune protection.

### 8.2. Maternal Antibody Levels and Vaccine-Induced Responses

Maternal antibody concentration is a key determinant of neonatal antibody levels at birth. Multiple studies have demonstrated strong correlations between maternal serum IgG titers and cord blood antibody concentrations, indicating that higher maternal antibody levels are associated with more efficient transplacental transfer [24,44,53]. These observations explain why maternal vaccination strategies are designed to increase maternal antibody concentrations during pregnancy and maximize neonatal antibody levels at birth.

Clinical trials of maternal RSV vaccination have demonstrated robust immunogenicity and efficient antibody transfer. In a large phase 3 randomized controlled trial, maternal immunization with a prefusion F protein-based RSV vaccine significantly increased maternal neutralizing antibody titers and corresponding cord blood antibody levels, resulting in reduced RSV-associated lower respiratory tract disease and hospitalization in infants [18]. Similarly, earlier trials of RSV F protein vaccines have shown that vaccination during pregnancy induces strong humoral responses that are efficiently transferred to the fetus [47,48]. Consistent findings have also been reported in maternal vaccination studies against other pathogens, such as group B streptococcus, further supporting the safety, immunogenicity, and effective transplacental transfer of vaccine-induced antibodies during pregnancy [58].

The timing of vaccine-induced antibody responses is an important factor for effective transplacental transfer. Following immunization, B-cell activation, germinal center formation, and affinity maturation require time to generate high-affinity IgG antibodies. Vaccination administered shortly before delivery may limit both antibody maturation and the time available for placental transfer, resulting in lower neonatal antibody concentrations.

### 8.3. Interval Between Vaccination and Delivery

Recent studies highlight the importance of the interval between maternal vaccination and delivery in determining antibody transfer efficiency. Longer intervals between vaccination and birth have been associated with higher cord blood antibody concentrations and improved cord-to-maternal transfer ratios [59].

In a prospective cohort study, vaccination occurring more than five weeks prior to delivery resulted in significantly higher transfer ratios compared with vaccination administered closer to delivery [59]. These findings suggest that both adequate time for antibody generation and sufficient duration for FcRn-mediated transplacental transport are required to maximize neonatal antibody levels.

Accordingly, current maternal RSV vaccination strategies generally target late pregnancy, typically between approximately 32 and 36 weeks of gestation, balancing optimal antibody induction with efficient placental transfer prior to delivery [18,59].

### 8.4. Additional Factors Influencing Antibody Transfer

In addition to gestational timing and maternal antibody levels, multiple maternal and placental factors influence transplacental antibody transfer efficiency. These include prematurity, low birthweight, maternal infections, placental inflammation, and systemic immunological conditions [24,41].

Maternal hypergammaglobulinemia has been associated with reduced transfer efficiency, potentially due to saturation of FcRn-mediated transport pathways [40]. Similarly, infections such as HIV or placental malaria have been shown to impair IgG transfer, likely through disruption of placental structure and function [37,41].

Placental inflammation and pathological changes may further alter FcRn expression and intracellular trafficking pathways, thereby affecting antibody transport [24]. In addition, maternal immunological history, including prior RSV exposure or vaccination, may influence the magnitude, affinity, and subclass distribution of antibody responses, with downstream effects on transfer efficiency.

Emerging evidence also suggests that structural characteristics of antibodies, including Fc glycosylation patterns, may influence Fc receptor interactions and contribute to variability in placental transfer [15,24]. Collectively, these maternal and placental factors illustrate the complexity of antibody transfer and explain part of the variability observed among pregnancies.

### 8.5. Implications for Maternal Immunization Strategies

Several mechanistic observations have direct implications for maternal RSV immunization strategies. The predominance of IgG1 responses following prefusion F vaccination is particularly advantageous because IgG1 is the subclass most efficiently transported across the placenta [14,18,24]. This supports vaccination during late pregnancy, when placental transport capacity is greatest and neonatal antibody acquisition is maximized [14,16,18,24,33,59].

The possibility that Fc glycosylation influences Fcγ receptor engagement and antibody function has also attracted considerable interest. Although current evidence remains incomplete, these findings raise the possibility that future vaccine formulations or adjuvants could be designed to promote antibody profiles with enhanced functional properties in addition to high neutralizing activity.

Preterm infants represent a particular challenge because shortened gestation limits the duration of placental antibody transfer [24,36,37,38,39]. Consequently, long-acting monoclonal antibodies remain an important complementary preventive option for infants who are unlikely to acquire sufficient maternally derived antibodies before birth [2,3,17,19,20].

Finally, ongoing advances in systems serology and structural immunology are expected to improve our understanding of the antibody characteristics associated with neonatal protection. Identifying these features may support the development of next-generation maternal vaccines and further refine immunization strategies aimed at extending passive protection during early infancy.

## 9. Maternal Vaccination and Monoclonal Antibody Prophylaxis: Complementary Strategies for RSV Prevention

Recent advances in RSV prevention have led to the development of two major immunological strategies for protecting infants during early life: maternal vaccination during pregnancy and passive immunization through long-acting monoclonal antibodies administered directly to infants. Both approaches aim to reduce the incidence of RSV-associated lower respiratory tract infection during a period of heightened vulnerability, when neonatal immune responses remain functionally immature and adaptive immunity is not fully established [2,3,5,30].

Maternal vaccination provides indirect protection by inducing antigen-specific humoral immune responses in pregnant individuals. Following immunization, antigen-specific B-cell activation and germinal center maturation lead to the production of high-affinity IgG antibodies directed against viral surface antigens, particularly the prefusion conformation of the RSV fusion (F) glycoprotein [8,18]. These vaccine-induced antibodies are subsequently transferred to the fetus via FcRn-mediated transplacental transport, resulting in systemic passive immunity at birth [13,16,24]. Preclinical and clinical studies have demonstrated that maternal immunization leads to substantial increases in RSV-specific antibody titers in both maternal and neonatal circulation, providing a biological basis for this preventive approach [34,35,39].

Clinical evidence further supports the effectiveness of maternal vaccination. In the phase 3 MATISSE trial, maternal immunization with a bivalent prefusion F RSV vaccine significantly reduced medically attended RSV-associated lower respiratory tract illness and hospitalization in infants during the first months of life [18]. Similarly, a phase 3 study evaluating the RSVPreF3-Mat vaccine demonstrated robust induction of neutralizing antibodies and efficient transplacental transfer to infants [60]. These findings further support the capacity of maternal RSV vaccination to generate and transfer RSV-specific antibodies to the infant.

A key mechanistic distinction between maternal vaccination and monoclonal antibody prophylaxis lies in the diversity and functional complexity of the antibody response. Maternal vaccination induces a polyclonal antibody repertoire comprising antibodies that recognize multiple antigenic epitopes and exhibit heterogeneous Fc structural characteristics, including variations in subclass distribution and glycosylation [21,25]. Experimental and systems serology studies suggest that this diversity may facilitate engagement of multiple Fc-dependent immune pathways, including antibody-dependent cellular cytotoxicity (ADCC), antibody-dependent cellular phagocytosis (ADCP), and complement activation [22,23]. However, although these mechanisms are biologically plausible and supported by preclinical and observational evidence, their precise contribution to the clinical protection observed following maternal RSV vaccination has not yet been fully established and requires further mechanistic investigation [21,22,23,24,25].

In contrast, monoclonal antibodies are epitope-specific and functionally uniform, providing highly potent neutralizing activity against a single antigenic site with a well-defined mechanism of action. Fc engineering can enhance pharmacokinetic properties, including FcRn binding and antibody half-life, thereby prolonging protection throughout a single RSV season [13,17]. Unlike vaccine-induced polyclonal responses, monoclonal antibodies do not rely on maternal immune activation or placental antibody transfer and provide immediate passive protection directly to the infant.

Despite these mechanistic differences, both strategies have demonstrated strong clinical effectiveness in reducing RSV-associated morbidity. Evidence from randomized clinical trials and real-world studies has consistently shown reductions in RSV-associated hospitalization following both maternal vaccination and monoclonal antibody administration [17,18,19]. Although recent comparative studies have suggested higher effectiveness of long-acting monoclonal antibodies in certain clinical settings [19,20], both interventions represent highly effective preventive strategies and should be considered within the context of individual clinical circumstances and national immunization policies.

Both preventive strategies have demonstrated favorable safety profiles in randomized clinical trials and post-marketing experience. Maternal RSV vaccination has generally been well tolerated, with adverse events consisting predominantly of mild to moderate local and systemic reactions, while the incidence of serious maternal or neonatal adverse events has been comparable to that observed in placebo groups [18,60]. Likewise, nirsevimab has demonstrated an excellent safety profile in both clinical trials and real-world studies, with serious adverse events occurring infrequently and the most commonly reported events including mild injection-site reactions and transient rash or hypersensitivity reactions [17,19]. Overall, current evidence supports the favorable benefit–risk profile of both interventions within their approved indications, with the choice of strategy guided primarily by clinical context, national recommendations, and individual patient characteristics [17,18,19].

Although maternal RSV vaccination and long-acting monoclonal antibodies have both demonstrated substantial clinical efficacy and ultimately provide passive antibody-mediated protection to the infant, they differ in the origin, generation, and delivery of the protective antibodies [17,18,61]. Whereas monoclonal antibodies provide a predefined antibody directly to the infant, maternal vaccination depends on maternal immune activation, vaccine-induced polyclonal antibody production, and subsequent transplacental transfer to the fetus [13,18,24]. Understanding these interconnected biological processes is essential for optimizing maternal immunization strategies and identifying reliable immunological correlates of neonatal protection [62].

Beyond transplacental antibody transfer, maternal vaccination may offer additional immunological and public health advantages. Breastfeeding represents a potential complementary pathway of passive immune protection, as breast milk contains RSV-specific antibodies together with cytokines, immune cells, extracellular vesicles, human milk oligosaccharides, and other bioactive factors that support mucosal immunity and early immune development [43,63]. Higher levels of RSV-specific antibodies in breast milk have been associated with a reduced risk of RSV-associated illness in infants; however, the extent to which maternal RSV vaccination modifies breast milk antibody composition and whether this translates into clinically meaningful protection remain uncertain and require further investigation [43]. Furthermore, maternal immunization actively engages pregnant individuals in protecting their infants from birth while facilitating integration into routine antenatal care, representing a unique preventive strategy that extends beyond passive antibody administration [12,13,14].

These approaches should be viewed as complementary. Maternal vaccination provides immediate protection at birth through transplacentally transferred antibodies, whereas monoclonal antibodies offer an alternative or additional protective strategy for infants who did not receive sufficient maternal antibody transfer, including preterm infants or those born to unvaccinated mothers. The complementary implementation of these strategies allows preventive approaches to be tailored according to maternal vaccination status, gestational age at birth, local recommendations, and individual clinical circumstances, thereby maximizing population-level protection against RSV [2,3,19,30].

In real-world clinical practice, the choice between maternal RSV vaccination and long-acting monoclonal antibodies should be individualized according to maternal vaccination status, gestational age at delivery, local immunization policies, product availability, and individual infant risk factors. Maternal vaccination is particularly well suited to pregnancies in which vaccination can be administered sufficiently before delivery to allow optimal antibody production and placental transfer, whereas long-acting monoclonal antibodies play a critical role for infants born to unvaccinated mothers, those delivered before adequate transplacental antibody transfer can occur, or when maternal vaccination is contraindicated or unavailable. Consequently, these approaches should be regarded as complementary rather than competing strategies, allowing flexible implementation to maximize protection against severe RSV disease across diverse clinical settings [2,3,17,18,19]. The key differences and complementary features between maternal vaccination and monoclonal antibody prophylaxis are summarized in Table 3.

## 10. Future Directions and Knowledge Gaps in Maternal RSV Immunization

Despite substantial advances in RSV vaccine development and the recent implementation of maternal immunization strategies, key gaps remain in understanding the immunological determinants of neonatal protection. Although maternal vaccination has been shown to significantly reduce RSV-associated lower respiratory tract disease in early infancy, the precise correlates of protection and the molecular mechanisms governing antibody transfer, persistence, and function are not yet fully defined [2,18]. Addressing these questions will improve our understanding of neonatal immunity and support the refinement of future maternal immunization strategies.

### 10.1. Defining Correlates of Protection for RSV in Early Life

A major challenge in RSV immunology is the lack of universally accepted correlates of protection. While neutralizing antibody titers are widely used as surrogate markers, accumulating evidence suggests that protection against RSV involves a combination of neutralizing activity and Fc-mediated effector functions [5,24].

Systems serology studies have identified antibody features—including Fcγ receptor binding profiles, IgG subclass distribution, and Fc glycosylation patterns—that are associated with antiviral immune responses and may influence clinical outcomes [16,21]. In neonates, whose immune systems rely heavily on maternally derived antibodies, these qualitative antibody characteristics may be particularly important.

Future studies integrating functional antibody profiling, systems serology, and clinical endpoints will be essential to define robust correlates of protection in early life. Such approaches may enable identification of composite immune signatures that better predict protection than neutralizing antibody titers alone.

### 10.2. Role of Antibody Glycosylation in Vaccine-Induced Immunity

Fc glycosylation is increasingly recognized as an important regulator of antibody function and may also contribute to differences in placental antibody transfer. Glycan composition modulates Fc receptor interactions, complement activation, and antibody structural conformation, thereby shaping effector function [15,21,24].

Recent studies suggest that antibodies enriched in specific glycan structures, such as digalactosylated forms, may be associated with enhanced Fc receptor engagement and may be preferentially transferred across the placenta [25]. However, the extent to which maternal vaccination modulates Fc glycosylation profiles—and how these changes influence neonatal protection—remains incompletely understood.

Future research integrating glycoproteomics, structural immunology, and maternal vaccination studies will be necessary to determine how vaccine-induced glycosylation patterns influence both FcRn-mediated transport and Fc-dependent immune function in neonates.

### 10.3. Systems Serology and Functional Antibody Profiling

Advances in high-dimensional immunological profiling have enabled comprehensive characterization of antibody responses through systems serology approaches. These methods allow simultaneous assessment of antibody subclasses, Fc receptor interactions, glycosylation patterns, and effector functions, providing a multidimensional view of humoral immunity [16,21].

Application of systems serology to maternal vaccination studies has the potential to identify functional antibody signatures associated with efficient placental transfer and neonatal protection. These approaches may identify previously unrecognized antibody characteristics associated with efficient placental transfer and neonatal protection.

### 10.4. Optimizing Vaccine Design for Maternal Immunization

While structural vaccinology has enabled major advances in RSV vaccine development, particularly through stabilization of the prefusion F protein [6,8], further optimization is needed to tailor vaccines for maternal immunization.

Further refinement of maternal RSV vaccines may focus not only on maximizing neutralizing antibody titers but also on optimizing antibody quality, including subclass distribution and Fc glycosylation patterns. Induction of IgG1-dominant responses may enhance both FcRn-mediated placental transfer and Fcγ receptor engagement [14,24]. In addition, vaccine platforms and adjuvants may influence glycosylation profiles, thereby modulating antibody functionality [21].

Understanding how vaccine design parameters—including antigen structure, adjuvant selection, and immunization timing—affect antibody structure and function will be essential for the development of next-generation maternal vaccines.

### 10.5. Integrating Maternal Vaccination with Infant Immunization Strategies

An additional priority is to define how maternal immunization can be integrated with other preventive strategies, including long-acting monoclonal antibodies and future infant vaccination programs. Maternal antibodies can influence infant immune responses through mechanisms such as epitope masking, antigen clearance, or immune complex formation, which may affect vaccine responsiveness in early life [11,30].

Understanding the interplay between maternally derived antibodies and infant immune development will be critical for designing coordinated immunization schedules that maximize protection while preserving the efficacy of infant vaccines. Such integrated approaches may be particularly important in optimizing protection against RSV across different risk groups, including preterm infants and those with limited maternal antibody transfer.

Continued progress in molecular immunology, systems biology, and maternal–fetal medicine is expected to refine maternal RSV immunization strategies and improve our understanding of neonatal immune protection. Advances in Fc receptor biology, antibody glycosylation, and high-dimensional immune profiling will likely contribute to the development of more effective preventive approaches for RSV and other infections during early life.

## 11. Limitations

This narrative review has several limitations that should be considered when interpreting the findings. First, the study selection process was not conducted within a formal systematic review framework, and no quantitative synthesis or meta-analysis was performed. Although a structured search strategy was applied, the absence of a formal risk-of-bias assessment may introduce selection bias and limit reproducibility.

Second, the available evidence on maternal RSV immunization remains heterogeneous, encompassing clinical trials, observational studies, and experimental models. Variability in study design, population characteristics, vaccination timing, and outcome definitions limits direct comparability across studies and complicates interpretation of results [18,48].

Third, several of the proposed immune mechanisms underlying neonatal protection following maternal RSV vaccination remain incompletely characterized [18]. While the protective role of neutralizing antibodies is well established, evidence supporting qualitative antibody features—including IgG subclass distribution, Fc glycosylation, and Fc-mediated effector functions—derives largely from systems serology studies, experimental models, and observational data rather than direct mechanistic clinical investigations [21,24,25]. Consequently, although these mechanisms are biologically plausible, further clinical and translational studies are needed to clarify their contribution to the protection observed following maternal RSV vaccination [21,24,25].

Finally, population-specific factors—including prematurity, maternal comorbidities, and geographical variability in infectious disease burden—may influence both antibody transfer efficiency and vaccine effectiveness. These variables are not consistently represented in the literature and may therefore limit the generalizability of current findings [38,41].

## 12. Conclusions

Maternal RSV vaccination has become an effective strategy for protecting infants during the first months of life by inducing maternal immune responses and enabling the transplacental transfer of protective antibodies. Together with long-acting monoclonal antibodies, it forms part of a complementary RSV prevention strategy that can be tailored according to maternal vaccination status, gestational age at birth, and individual clinical circumstances. Neutralizing antibodies remain the best-established correlate of protection, whereas qualitative antibody characteristics—including Fc receptor interactions and Fc glycosylation—may also contribute to neonatal immunity. Nevertheless, the clinical relevance of these Fc-mediated mechanisms has yet to be fully defined. Integrating mechanistic immunology with clinical and real-world effectiveness data will help define reliable correlates of protection, refine maternal immunization strategies, and optimize RSV prevention during early infancy.

## Figures and Tables

**Table 1:** Determinants of Transplacental Antibody Transfer.

Factor	Effect on Transfer	Mechanism	Key References
Gestational age	↑ with advancing gestation	↑ FcRn expression and placental surface area	[14,24,51,52]
Maternal antibody levels	↑ transfer with higher titers	Concentration gradient	[44,53]
IgG subclass	IgG1 > IgG3/4 > IgG2	FcRn affinity differences	[4]
Fc glycosylation	Modulates transfer	Fc structure & receptor interaction	[22,54]
Maternal infections	↓ transfer	Placental dysfunction	[37,41]
Prematurity	↓ transfer	Reduced exposure time	[38]

Abbreviations: FcRn, neonatal Fc receptor; IgG, immunoglobulin G; ↑, increased; ↓, reduced.

**Table 2:** Fc Glycosylation Modifications and Functional Effects.

Glycan Modification	Effect on Fc Function	Immunological Outcome
Afucosylation	↑ FcγRIIIa binding	↑ ADCC
Galactosylation	↑ C1q binding	↑ Complement activation
Sialylation	Anti-inflammatory	Immune regulation
Bisecting GlcNAc	Modulates Fc structure	Altered receptor binding

Abbreviations: ADCC, antibody-dependent cellular cytotoxicity; FcγRIIIa, Fc gamma receptor IIIa; C1q, complement component 1q; GlcNAc, N-acetylglucosamine; ↑, increased.

**Table 3:** Maternal RSV Vaccination versus Long-Acting Monoclonal Antibodies for Infant RSV Prevention.

Feature	Maternal RSV Vaccination	Long-Acting Monoclonal Antibodies (e.g., Nirsevimab)
Mechanism of protection	Indirect protection through transplacental transfer of vaccine-induced maternal IgG	Direct passive immunization administered to the infant
Target antigen	Prefusion F protein	Prefusion F protein
Antibody type	Polyclonal antibodies	Monoclonal antibody
Source of antibodies	Endogenously generated following maternal vaccination	Exogenously administered antibody
Timing of administration	During pregnancy (optimal administration according to current recommendations)	After birth (before or during the RSV season)
Onset of protection	At birth following placental antibody transfer	Within days after administration
Duration of protection	Primarily during the first months of life; declines with physiological maternal IgG waning	Approximately one RSV season owing to extended half-life
Fc-mediated effector functions	Potential for diverse Fc-mediated functions (ADCC, ADCP, complement activation); clinical contribution remains under investigation	Epitope-specific neutralization; Fc-mediated functions depend on antibody engineering and are not the primary mechanism of protection
Antibody glycosylation	Fc glycosylation may influence placental transfer, antibody persistence, and effector functions; clinical relevance remains incompletely established	Defined glycosylation profile determined during manufacturing; limited biological variability
Clinical efficacy	Reduces RSV-associated lower respiratory tract disease and hospitalization during early infancy	Reduces RSV-associated lower respiratory tract disease and hospitalization during early infancy; recent real-world studies have reported higher effectiveness in some settings
Potential postnatal immune support through breastfeeding	May provide continued transfer of RSV-specific antibodies and other bioactive immune factors through breast milk; protective contribution requires further investigation	Not applicable
Maternal participation in infant protection	Active maternal immunization integrated into antenatal care, directly involving the mother in infant protection	Passive immunization delivered directly to the infant after birth
Main advantages	Protection begins at birth; polyclonal immune response; potential additional immune support through breastfeeding; integrated into routine antenatal care	Rapid protection independent of maternal vaccination status or placental antibody transfer; prolonged serum half-life
Main limitations	Effectiveness depends on vaccination timing, placental antibody transfer, gestational age, and maternal factors; vaccine uptake remains suboptimal in some settings	High cost, access and implementation challenges; requires administration to each infant
Safety profile	Generally well tolerated; predominantly mild local and systemic reactions	Generally well tolerated; mild injection-site reactions most common; serious adverse events uncommon

Abbreviations: IgG, immunoglobulin G; RSV, respiratory syncytial virus; ADCC, antibody-dependent cellular cytotoxicity; ADCP, antibody-dependent cellular phagocytosis.

## Data Availability

No new data were created or analyzed in this study. Data sharing is not applicable to this article.
